# Coexistence of Granular Cell Tumor and Invasive Ductal Breast Cancer in Contralateral Breasts: A Case Report

**DOI:** 10.3390/ijms150813166

**Published:** 2014-07-25

**Authors:** Maurizio Di Bonito, Monica Cantile, Francesca Collina, Rossella De Cecio, Teresa Petrosino, Gerardo Botti

**Affiliations:** 1 Pathology Unit, Department of Diagnostic Pathology and Laboratory Medicine, Istituto Nazionale Tumori “Fondazione Pascale” Istituti di Ricovero e Cura a Carattere Scientifico (IRCCS), Naples 80131, Italy; E-Mails: mauriziodibonito@libero.it (M.D.B.); francesca.collina@virgilio.it (F.C.); rosselladececio@libero.it (R.D.C.); g.botti@istitutotumori.na.it (G.B.); 2 Diagnostic Imaging Day Hospital Unit, Diagnostic Imaging, Radiation and Nuclear Oncology Department, Istituto Nazionale Tumori “Fondazione Pascale” Istituti di Ricovero e Cura a Carattere Scientifico (IRCCS), Naples 80131, Italy; E-Mail: t.petrosino@istitutotumori.na.it

**Keywords:** GCT, breast imaging, synchronous tumors

## Abstract

Granular cell tumor (GCT) is a benign tumor of the breast that can mimic, on breast imaging, invasive carcinomas. Biological evolution of mammary GCT is unknown, especially if it is associated with an invasive carcinoma in the same or contralateral breast. This report details the morphological features of these synchronous lesions highlighting their biological characteristics and suggesting an appropriate follow up.

## 1. Introduction

Granular cell tumour (GCT) of the breast is a relatively uncommon lesion, usually benign in nature.

In 1926, Abrikossoff described for first time this lesion, proposing a possible origin from smooth muscle cells [1]. Then, on the basis of its immunophenotypic and ultrastructural features, it was finally determined that it derives from perineural cells [2].

Clinically, GCT can mimic carcinoma, presenting as a palpable mass, and for its fibrous consistency. On mammographically and sonographically examination, GCT can present as not clearly defined lesions, similar to primary invasive carcinoma.

Histologically, GCTs arise from intra-lobular breast stroma creating cords that extend into adjacent normal breast parenchyma, a characteristic that simulates the growth pattern of invasive breast cancer carcinoma. Moreover, the main histologic element that distinguishes GCTs from other lesions is the presence of granular cytoplasm probably caused by accumulation of secretory granules, mitochondria, or lysosomes.

Several evidences reported the presence of synchronous or independent tumors, with different biological characteristics in contralateral breasts [3].

However, only few cases of coexistence of a benign lesion of the breast with invasive carcinoma was described in distinct breasts [4]. Only one case reported the co-localization of GCT and infiltrating ductal carcinoma in the same breast [5].

In this report we describe the coexistence of granular cell tumor of the breast, and an invasive ductal carcinoma in distinct breasts. The interest of this report is represented not only by rarity of this breast neoplasm and for the diagnostic difficulties to distinguish radiologically benign and malignant lesions, but also for limited knowledge on its biological trend especially if it was associated with other malignant tumors of the breast.

## 2. Case Report

**Figure 1:**
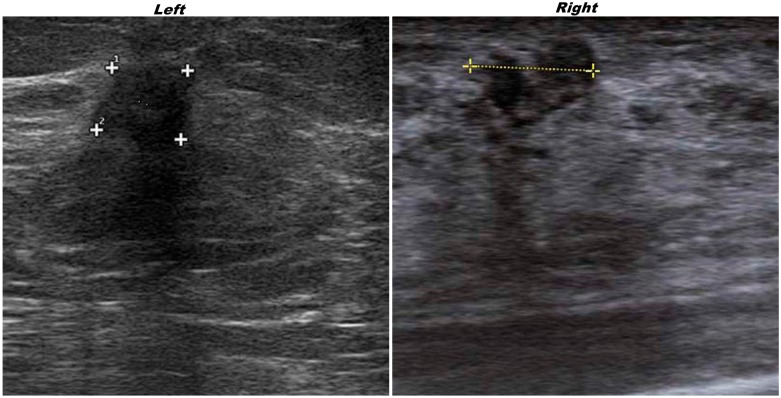
Ultrasonography of the **left** invasive ductal carcinoma(IDC) and **right** granular cell tumor (GCT) breast lesions. “Plus signs” underline the presence of suspicious lesions.

A 67-year-old woman, after a mammography with suspicious findings, was referred to our Institution. The ultrasonography revealed in the superior external quadrant of the left breast a solid hypoechoic mass of about 1.5 cm (Figure 1, on the left), suspicious for malignancy.

Ultrasonography on contralateral breast showed, in the upper inner quadrant, a solid hypoechoic oval mass of about 2 cm (Figure 1, on the right).

The patient underwent to definitive surgery for excision of all lesions.

**Figure 2:**
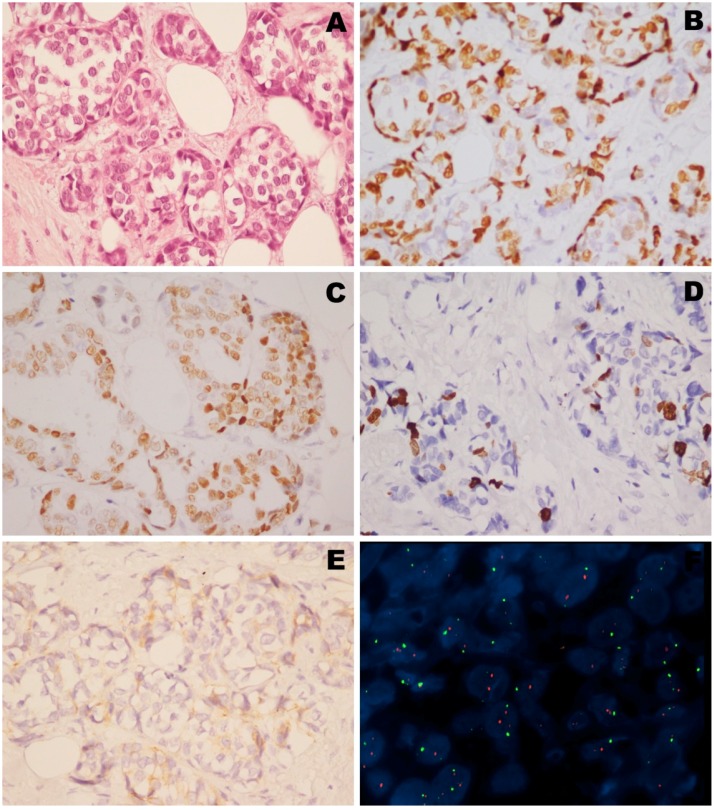
Immunophenotype of the left breast lesion: (**A**) H&E morphology (40×); (**B**) immunopositivity for ER (40×); (**C**) immunopositivity for PgR (40×), (**D**) immunopositivity for Ki67 (40×); (**E**) immunopositivity for HER-2 (40×). In the bottom right FISH analysis of *HER-2* gene without amplification signals. HER-2 probe spans the entire *HER-2* gene is labeled in SpectrumOrange. The CEP 17 probe is labeled in SpectrumGreen and hybridizes to the alpha satellite DNA located at the centromere of chromosome 17. Two HER-2 (red) and two CEP 17 (green) signals indicate no HER-2 amplification.

For the left nodule, histological evaluation confirmed the existence of an invasive ductal carcinoma (IDC) of intermediate grade of malignity. Immunohistochemical antibodies panel, carried out on the lesion, showed a strong positivity for ER (75%), and for PgR (75%), a high proliferation index ki67, a positivity for HER-2 with a 1+ score FDA (Figure 2). Further cytogenetic analysis, using the FISH method, did not show any gene amplification of HER-2 (Figure 2).

**Figure 3:**
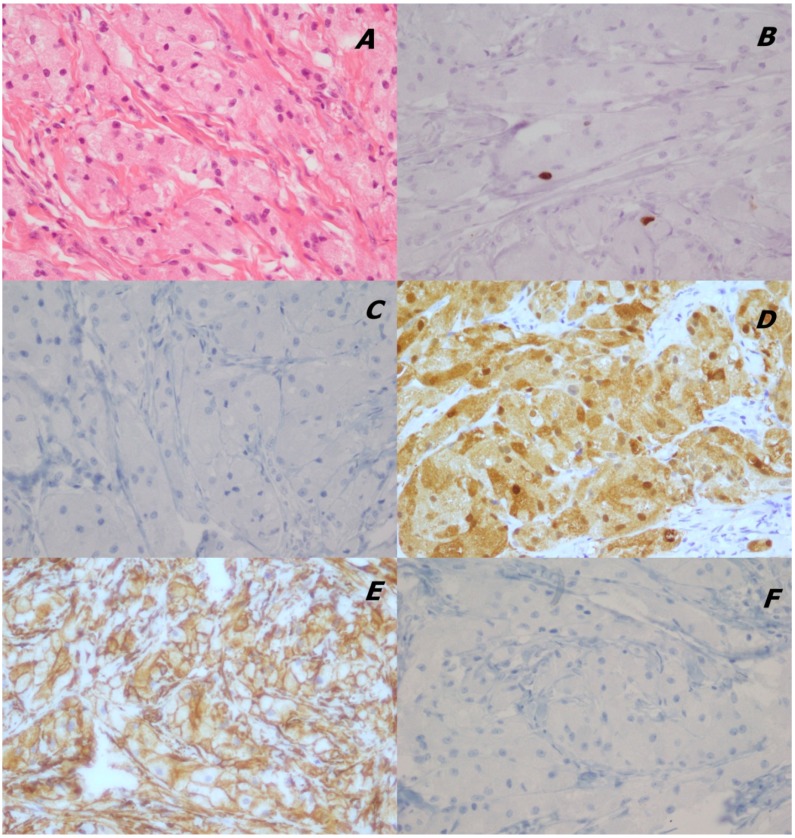
Immunophenotype of the right breast lesion: (**A**) H&E morphology (40×); (**B**) immunopositivity for Ki67 (40×); (**C**) immunonegativity for panCK (40×); (**D**) immunopositivity for vimentin (40×); (**E**) immunopositivity for S100 (40×); (**F**) immunonegativity for CD68 (40×).

For the right nodule, the histological evaluation and immunohistochemical antibodies panel showed a low proliferation index ki67, a positivity for vimentin and S100, and a negativity for pan CK and CD68 markers (Figure 3).

Morphology and immunoprofile were consistent with a diagnosis of GCT.

## 3. Discussion

Primary tumors that coexist at the time of diagnosis are defined “synchronous” and this condition is quite common for malignant lesions that occur in bilaterally symmetrical organs, such as breasts [3], kidneys [6], ovaries [7], and testes [8].

However, synchronous benign and malignant lesions in separate breasts have not been reported. In literature, only one case, showing the presence of a benign phylloides tumor and invasive ductal carcinoma in contralateral breast has been described [4] and one case reported the co-localization of GCT and invasive carcinoma in the same breast [5].

In this report we present the coexistence of a very rare lesion of the breast, a germinal cell tumor, and an invasive ductal carcinoma in distinct breasts.

GCTs are usually benign neoplasms that account for less than 1% of breast lesions. Malignant GCTs of the breast are rare in the literature [9]. GCTs have been shown to be non-dependent on estrogen and progesterone. For its similarities in ultrastructural features, these tumors are thought to originate from perineural cells, probably from a Schwann-cell of origin [2]. GCT of the breast accounts for only 6% of all GCTs reported.

In contrast to most other breast tumors, which occur predominantly in the upper outer quadrant, GCTs are most frequently found in the upper inner quadrant, but radiographically, it is impossible to establish a definitive diagnosis of GCT of the breast without a biopsy. Microscopic characteristics of GCT typically include aggregates of loosely infiltrating large round or polygonal cells with abundant eosinophilic granular cytoplasm and variable amounts of collagenous stroma. The nuclei are often small and centrally located.

By immunohistochemistry, S100 can be used for diagnosis, supporting neural origin, and distinguishing GCTs from other neoplasms with abundant eosinophilic granular cytoplasm, and CD68. Moreover, the negativity for cytokeratins rules out an adenocarcinoma diagnosis. A high proportion are also reactive for vimentin, which is not often detected in carcinomas, a helpful feature that aids in distinguishing between the two.

In our presented case, S100 and vimentin are strongly expressed, while pan CK and CD68 appear negative. The morphology, associated to this immunoprofile and a low proliferative index supported the GCT diagnosis.

The case described is interesting not only for the rarity of GCT lesion, but also for their association with a malignant lesion in contralateral breast.

Genetic predisposition to develop multiple cancers is still currently the subject of numerous studies. It was frequently reported that the coexistence of two or more tumors, synchronous or metachronous, in the same patient, may also be correlated with hereditary syndromes [10]. However a genetic relation between these lesions was not described, thus we can assume that their synchrony is purely casual.

## 4. Conclusions

GCT represents a rare lesion in the breast, and in the literature very little information is provided concerning its biological evolution. In fact, less than 1% of all GCTs, including mammary lesions, are malignant. For this reason, particularly if this lesion is associated with other malignant tumors in the same breast, but also in the contralateral, complete surgical excision is always recommended. Local recurrence has been reported [11] after incomplete excision; thus, patients must be subjected to an appropriate follow up.
